# Acute Cholecystitis Caused by Campylobacter jejuni Mimicking Acute Coronary Syndrome

**DOI:** 10.7759/cureus.53608

**Published:** 2024-02-05

**Authors:** Hiroki Uehara, Yutaro Oe, Takaki Yoshimura, Takahiro Gunji, Masaki Okuyama

**Affiliations:** 1 Cardiovascular Medicine, Kin-ikyo Chuo Hospital, Sapporo, JPN

**Keywords:** coronary angiography, ecg change, acute coronary syndrome, campylobacter jejuni, acute cholecystitis

## Abstract

Campylobacter spp. is a widely recognized pathogen accountable for acute enteritis, frequently linked to sepsis, primarily attributed to C. jejuni. Instances of Campylobacter-induced cholecystitis are infrequent, with only a limited number of documented case reports. Acute cholecystitis has been sporadically documented to induce electrocardiographic alterations, occasionally simulating an acute coronary syndrome (ACS). Herein, we present an instance of cholecystitis induced by C. jejuni, posing a challenge in its differentiation from ACS due to electrocardiographic modifications.

An 85-year-old Japanese male presented to our hospital with a complaint of chest discomfort lasting one hour. His medical history included hypertension, dyslipidemia, and effort angina pectoris, with a prior percutaneous coronary intervention. The chest discomfort, accompanied by pain and pressure, raised uncertainty about its similarity to a previous angina episode. Vital signs were in the normal range. Physical examination revealed no abnormal heart or lung sounds. Electrocardiography indicated a right bundle branch block and new ST-segment elevation in V2-3. Echocardiography, chest X-rays, and blood tests showed no abnormalities. Emergency coronary angiography revealed no stenosis. Post-angiography, chest discomfort persisted, and the patient developed fever and chills. Contrast-enhanced CT revealed gallbladder lithiasis, prompting suspicion of sepsis. C. jejuni was detected, and antimicrobial therapy resolved symptoms.

## Introduction

Campylobacter spp. is a well-known pathogen responsible for acute enteritis, and it is often associated with sepsis, due mainly to C. jejuni [1]. Cases of Campylobacter causing cholecystitis are rare, and there are only a few case reports [2-7]. On the other hand, acute cholecystitis has been reported to rarely cause electrocardiographic changes, sometimes mimicking an acute coronary syndrome (ACS) [8-11].

We present a case of cholecystitis caused by C. jejuni that was difficult to distinguish from ACS due to electrocardiographic changes.

## Case presentation

An 85-year-old Japanese male presented to our hospital complaining of chest discomfort that lasted one hour. His medical history included hypertension, dyslipidemia, and effort angina pectoris (EAP), and he had undergone percutaneous coronary intervention (PCI). His chest discomfort was accompanied by pain and pressure, and it was not clear whether the symptoms were similar to those of a previous episode of angina pectoris. At his presentation, the vital signs were as follows: blood pressure 137/72 mmHg; heart rate 56 /min; respiratory rate 15/min; oxygen saturation on room air 96%; and temperature 36.0°C. The physical examination revealed no abnormal heart or lung sounds. There was no abdominal tenderness, and Murphy's sign was negative. Chest X-rays showed no cardiac enlargement or pulmonary congestion.

**Figure 1:**
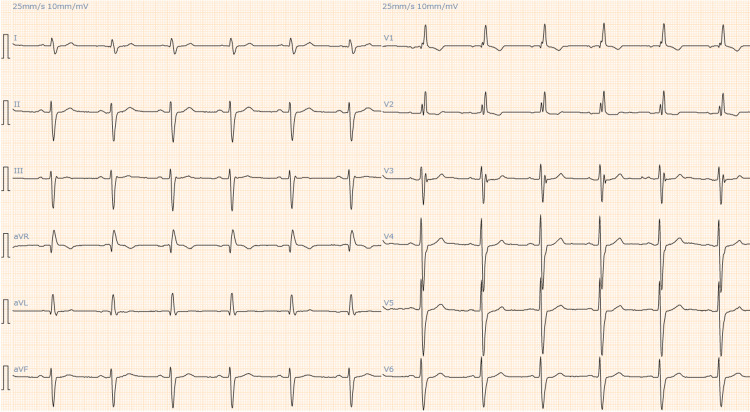
The patient's ECG three months before the present admission showed a right bundle branch block.

**Figure 2:**
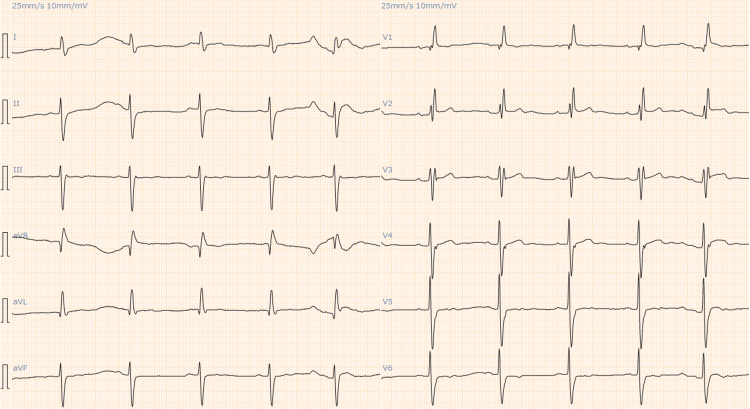
At presentation, ECG showed ST-segment elevation in lead V2–3, which was not seen previously.

Electrocardiography (ECG) showed a right bundle branch block as had been observed three months earlier, but it also showed ST-segment elevation in lead V2-3, which was not seen earlier (Figures 1, 2).

Echocardiography depicted normal left ventricular systolic function and no obvious wall motion impairment. The results of blood tests were all normal, as follows: white blood cell count 8.32×103/µL, serum total bilirubin 0.4 mg/dL, alkaline phosphatase 79 IU/L, aspartate aminotransferase 30 IU/L, alanine aminotransferase 19 IU/L, sodium 142 mmol/L, blood urea nitrogen 17.9 mg/dL, creatinine 0.99 mg/dL, creatinine kinase 84 IU/L, and troponin-T 0.014 ng/mL (Table 1).

**Table 1:** Results of blood tests WBC: White blood cell; PLT: platelet count; TP: total protein; CRP: C-reactive protein; AST: aspartate aminotransferase; ALT: alanine aminotransferase; ALP: alkaline phosphatase; LDH: lactate dehydrogenase; BUN: blood urea nitrogen; T-BIL: serum total bilirubin

Laboratory test	Value	Unit	Normal range
WBC	8320	/µL	4000-11000
Hb	14	g/dL	12-16
PLT	168	×10^9^/L	150-450
TP	6.4	g/dL	6.5-8.0
ALB	4.1	g/dL	4.0-5.2
CRP	0.11	mg/dL	0-0.3
AST	30	U/L	0-30
ALT	19	U/L	0-30
LDH	173	U/L	119-229
ALP	79	U/L	38-113
γ-GTP	34	mg/dL	0-50
T-BIL	0.4	mg/dL	0.2-1.2
BUN	17.9	mg/dL	7-24
Cre	0.99	mg/dL	0-1.0
CK	84	U/L	60-287
Troponin T	0.014	ng/mL	0-0.014

Because of the patient's chest symptoms with ST elevation on ECG, emergency coronary angiography was performed, but no stenosis was detected in the coronary arteries.

**Figure 3:**
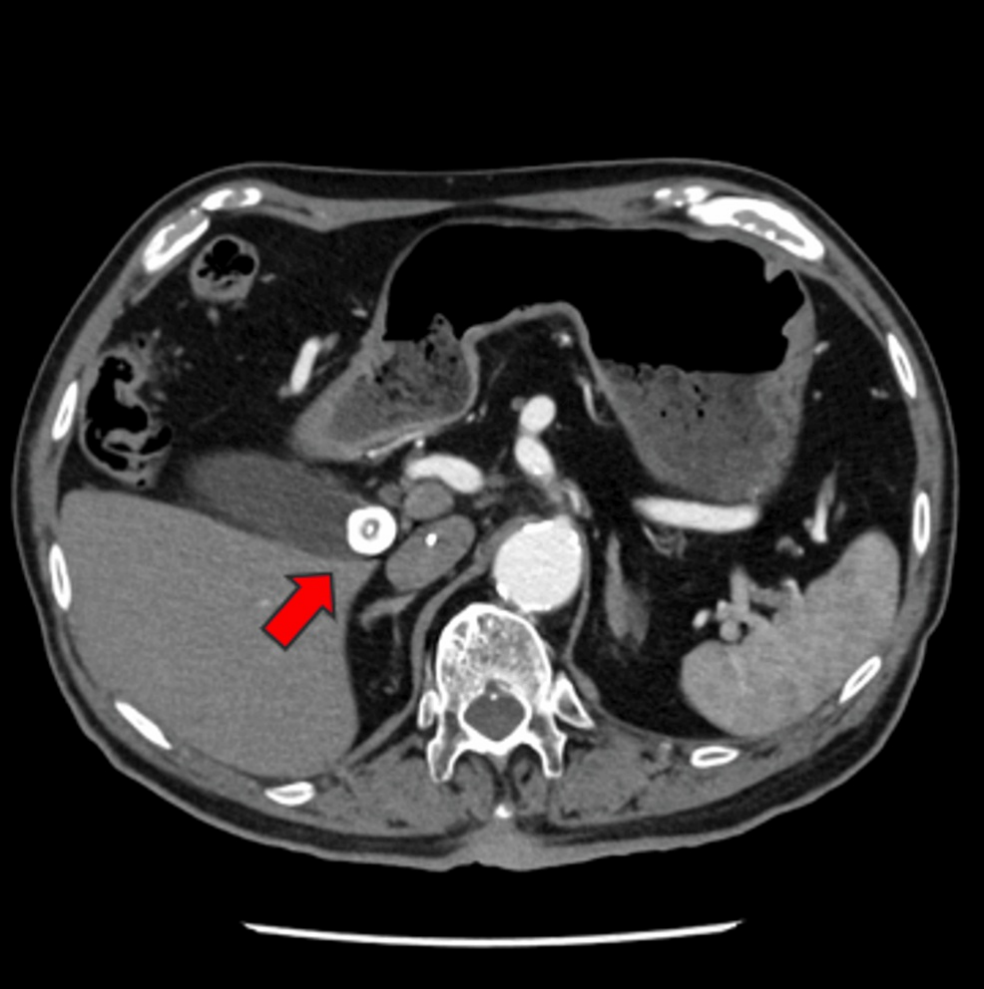
Contrast-enhanced CT performed the day after admission showed gallstones in the gallbladder neck (arrow).

Following coronary angiography, the patient's thoracic unease exhibited a propensity for amelioration, yet persisted. The patient was repeatedly checked for chest and abdominal tenderness, but no tenderness was noted. An upper gastrointestinal endoscopy was performed, revealing no remarkable findings. The day after admission, the patient had chills, shivers, and a fever reaching 39.1°C. Contrast-enhanced CT revealed lithiasis in the gallbladder but no evidence of gallbladder swelling or inflammation of the surrounding area (Figure 3).

Based on the suspicion of sepsis, ceftriaxone was initiated after two sets of blood cultures were taken.

**Figure 4:**
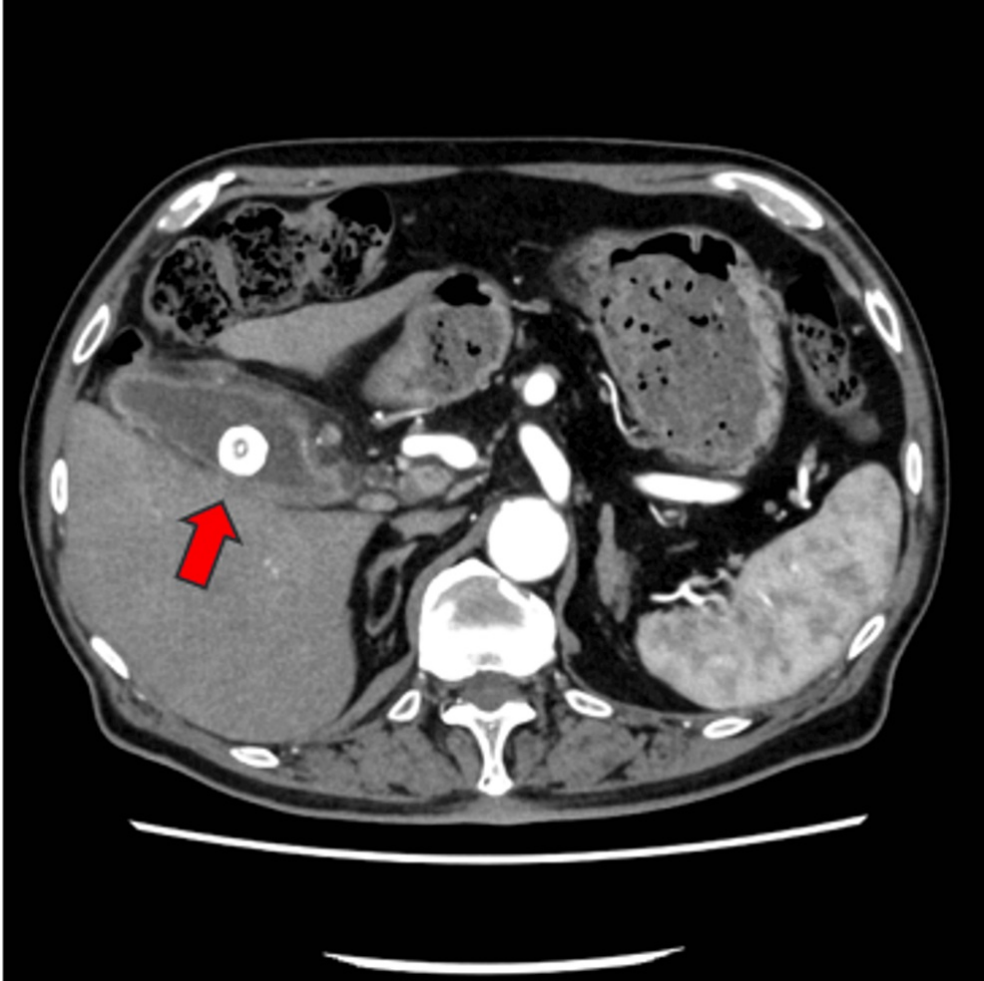
Compared to the position two days earlier, gallstones had moved from the neck of the gallbladder to the body (arrow).

**Figure 5:**
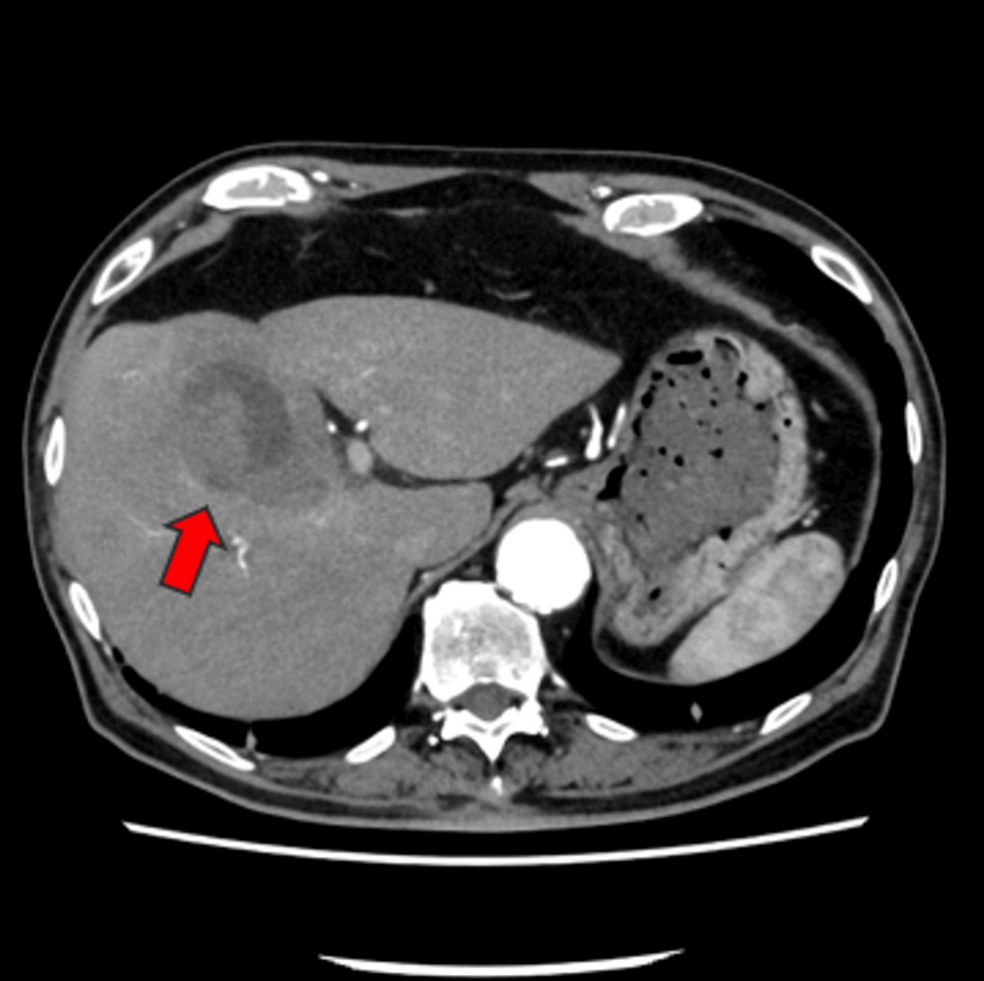
Contrast-enhanced CT on day 4 of hospitalization showed a swollen and edematous gallbladder (arrow).

By the second day of hospitalization, the patient's chest symptoms gradually moved to the upper abdomen, with exacerbations and remissions. An abdominal echo performed on the third hospitalization day showed that the gallbladder wall was swollen to approx. 6 mm. Additional contrast-enhanced CT was performed on the fourth hospitalization day, demonstrating that the gallstones had migrated from the neck of the gallbladder to the body. The gallbladder was swollen and edematous, and a complication of liver abscess around the gallbladder was also suspected (Figures 4, 5).

**Figure 6:**
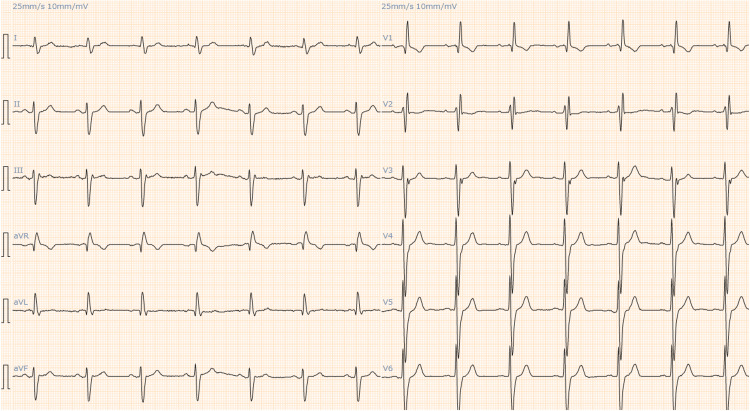
At day 4 after admission, ECG showed that the ST segment elevation had returned.

After antimicrobial therapy was initiated, the fever subsided and the chest and abdominal discomfort was no longer present. C. jejuni was detected in two of four sets of blood cultures taken one week after admission. Ceftriaxone was discontinued and the patient was started on azithromycin for three days and piperacillin tazobactam intravenously for one week. Four days after admission, the ECG returned to normal (Figure 6). 

He was discharged on the 17th hospitalization day. 

**Figure 7:**
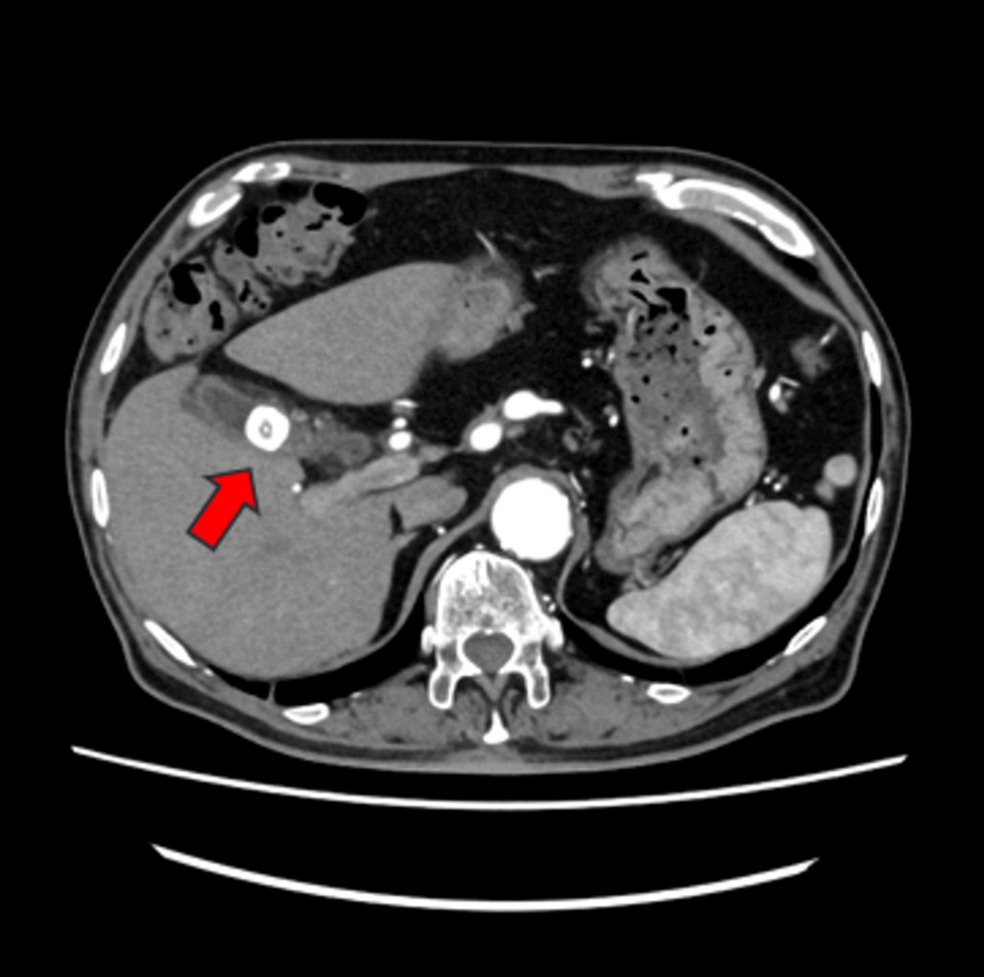
One month after discharge, contrast-enhanced CT revealed improvement in gallbladder swelling (arrow).

No chest or abdominal symptoms occurred after discharge. One month after the patient's discharge, a contrast-enhanced CT scan showed improvement in the gallbladder swelling and edematous changes (Figure 7). Gallstones remained, and we recommended a standby cholecystectomy, but the patient did not wish to undergo surgery.

## Discussion

This is a very rare and instructive case of cholecystitis caused by Campylobacter which mimics ACS. Acute cholecystitis due to Campylobacter was first reported in 1979 [2], and there have been several reports since then [3-7]. Some cases have been reported in which gastroenteritis symptoms such as diarrhea preceded the onset of cholecystitis due to Campylobacter [2,4], but many cases were not accompanied by diarrhea. Udayakumar et al. reviewed 14 cases of cholecystitis caused by Campylobacter, including their own patient; only five of the 14 patients had diarrhea [4].

In general, antimicrobials are often not needed for enteritis caused by Campylobacter, but in many cases of cholecystitis, macrolide antimicrobials have been administered. Dakdouki et al. similarly summarized 14 cases of cholecystitis caused by Campylobacter, including their own patient, and they confirmed that all had symptoms of abdominal pain [5]. Several routes of transmission of Campylobacter spp. to humans have been recognized, due mainly to direct contact with the milk or parts of animals, the ingestion of contaminated milk or foods of animal origin, and the ingestion of contaminated water. The diagnosis of Campylobacter cholecystitis is usually missed because a Campylobacter culture is not routinely requested after a cholecystectomy or drainage of bile [4-6].

In our patient's case, because his symptoms improved with conservative treatment, cholecystectomy and cholecystectomy drainage were not performed. No bile culture was performed, but (i) CT findings demonstrated acute cholecystitis, (ii) no other infected organs were detected, and (iii) there was no diarrhea; the diagnosis of acute cholecystitis and bacteremia due to C. jejuni was thus made.

Another important finding in this case was the complication of electrocardiographic changes. Several cases of cholecystitis with ST changes on ECG have been reported [8-11]. Aksay et al. described cholecystitis with transient T-wave changes [8]. Durning et al. also reported a case of acute cholecystitis with transient ST-segment elevation, and the ST-segment elevation improved within five hours [9], indicating that ECG changes caused by acute cholecystitis can be transient and reversible.

Ozeki et al. identified 16 patients (among 5,552 patients examined at a cardiology department during the period from June 2010 to June 2014) who were diagnosed with acute cholecystitis during their hospitalization [11]. Eleven of the 16 patients had developed cholecystitis while hospitalized for cardiovascular disease. Interestingly, five of the 16 patients were initially diagnosed with cardiac conditions but were eventually shown to have acute cholecystitis. Four of those five patients complained of chest pain, and thus ACS was suspected; the cases of two of the four patients were accompanied by ST changes in ECG, and all four patients had negative blood troponin T.

There are several possible mechanisms of ECG changes caused by acute cholecystitis: (i) distention of the bile duct may reduce the coronary artery flow; (ii) there may be a coronary vasospasm caused by a vagally mediated reflex; and (iii) ECG changes may also be evoked in response to inflammation of other visceral organs (11). In our patient's case, coronary angiography was performed early in the disease onset when ECG changes were occurring, but no obvious abnormalities of blood flow were identified. At the time of that visit, there was no inflammatory spillover to the gallbladder periphery on CT scans. The patient's chest discomfort on arrival was probably due to gallstone colic, and we suspect that the cholecystitis developed when chills and shivers appeared the day after admission. The mechanism underlying the ECG changes in this patient's cholecystitis is not clear, but it is possible that microcirculatory disturbances were involved due to pain.

When a patient comes in with epigastric pain with ECG changes, we would mistake it for ACS. Coronary angiography should not be performed in patients with acute cholecystitis, and appropriate treatment should be given early.

## Conclusions

This case describes a rare instance of C. jejuni-induced cholecystitis mimicking ACS. Electrocardiographic changes added complexity to the diagnosis, highlighting the need for considering atypical presentations in ACS evaluation. The patient's symptoms improved with conservative treatment, revealing the importance of a nuanced approach in distinguishing gastrointestinal and cardiac conditions.
